# Molecular Profiling of Benign Metastasizing Leiomyoma of the Uterus Revealing Unique Novel Therapeutic Targets

**DOI:** 10.7759/cureus.7701

**Published:** 2020-04-16

**Authors:** Dawood Findakly, Jue Wang

**Affiliations:** 1 Internal Medicine, Creighton University Arizona Health Education Alliance/Valleywise Health Medical Center, Phoenix, USA; 2 Genitourinary Oncology, Creighton University School of Medicine/University of Arizona Cancer Center at Dignity Health St. Joseph’s, Phoenix, USA

**Keywords:** benign metastasizing leiomyoma (bml), intravascular leiomyomatosis (ivl), uterine leiomyoma, molecular analysis

## Abstract

Extra-uterine manifestations of benign uterine leiomyoma (fibroids) are rare. Benign metastasizing leiomyoma (BML) comprises an uncommon variant characterized by metastatic lung nodules. The pathologic characteristics for BML are well known in the literature; however, the underlying biology and molecular mechanisms remain poorly understood.

We present a case of a 43-year-old woman who presented to the hospital complaining of dyspnea and lower extremity edema. Medical history includes a previous hysterectomy for leiomyomata two years prior. A reduced ejection fraction and right atrium globular filling defect are seen on transthoracic echo (TTE). CT scans of the chest, abdomen, and pelvis reported pelvic mass with an extensive inferior vena cava (IVC) thrombus extending into the right atrium, which was subsequently completely resected. Subsequent histopathology for the thrombus reported intravascular leiomyomatosis (IVL) and pelvic mass reported benign leiomyoma. Two years later, the symptoms recurred, and a chest CT revealed new pulmonary nodules. Subsequent pathology from a biopsy of these nodules was consistent with BML with ER+/PR+ on immunohistochemical staining.

Genetic testing showed amplification of JUN, cyclin-dependent kinase 4 (CDK4), and MCL1, and loss of SUFU, AT-rich interaction domain 1A (AR1D1A), RB transcriptional corepressor 1 (RB1), and hepatocyte nuclear factor 1-alpha (HNF1A). The patient deemed to be a poor surgical candidate, and, therefore, she was started on hormonal treatment with leuprolide and letrozole. The disease remained stable upon follow-up at 48 months. Here, we report novel genomic profiling findings for the first time in a patient with a newly diagnosed BML. These findings may suggest molecular evidence that IVL may not be as benign as previously thought.

Our study further highlights the value of genetic profiling in the understanding of this tumor's behavior and identification of new patient-specific therapeutic targets.

## Introduction

Benign metastasizing leiomyoma (BML) is an uncommon condition with approximately 200 cases reported in the literature since the case first described by Steiner in 1939 [1]. It was most commonly incidentally diagnosed in middle-aged women several years after uterine leiomyoma surgery. Total hysterectomy is the most common type of uterine surgery to precede BML diagnosis. Moreover, lungs are the most common extrauterine site of spread at the time of diagnosis [2-4]. 

Our current understanding of this condition is limited to the cytogenetic level. Novel biomarkers have the potential to help risk-stratify patients with BML, thus enabling the development of a novel and precise molecular-guided therapeutic approach to management [5]. Here we present a case of a 43-year-old woman with BML and intravascular leiomyomatosis (IVL) where the molecular profiling of BML suggests molecular evidence for a malignant potential of this previously thought benign disease.

## Case presentation

A 43-year-old Hispanic woman who had a past medical history relating to hypertension, obesity, and stroke was first admitted to the hospital in December 2014. There she had undergone hysterectomy for abnormal uterine bleeding, and surgical pathology, and at that time, was confirmed as having uterine leiomyoma. 

**Figure 1:**
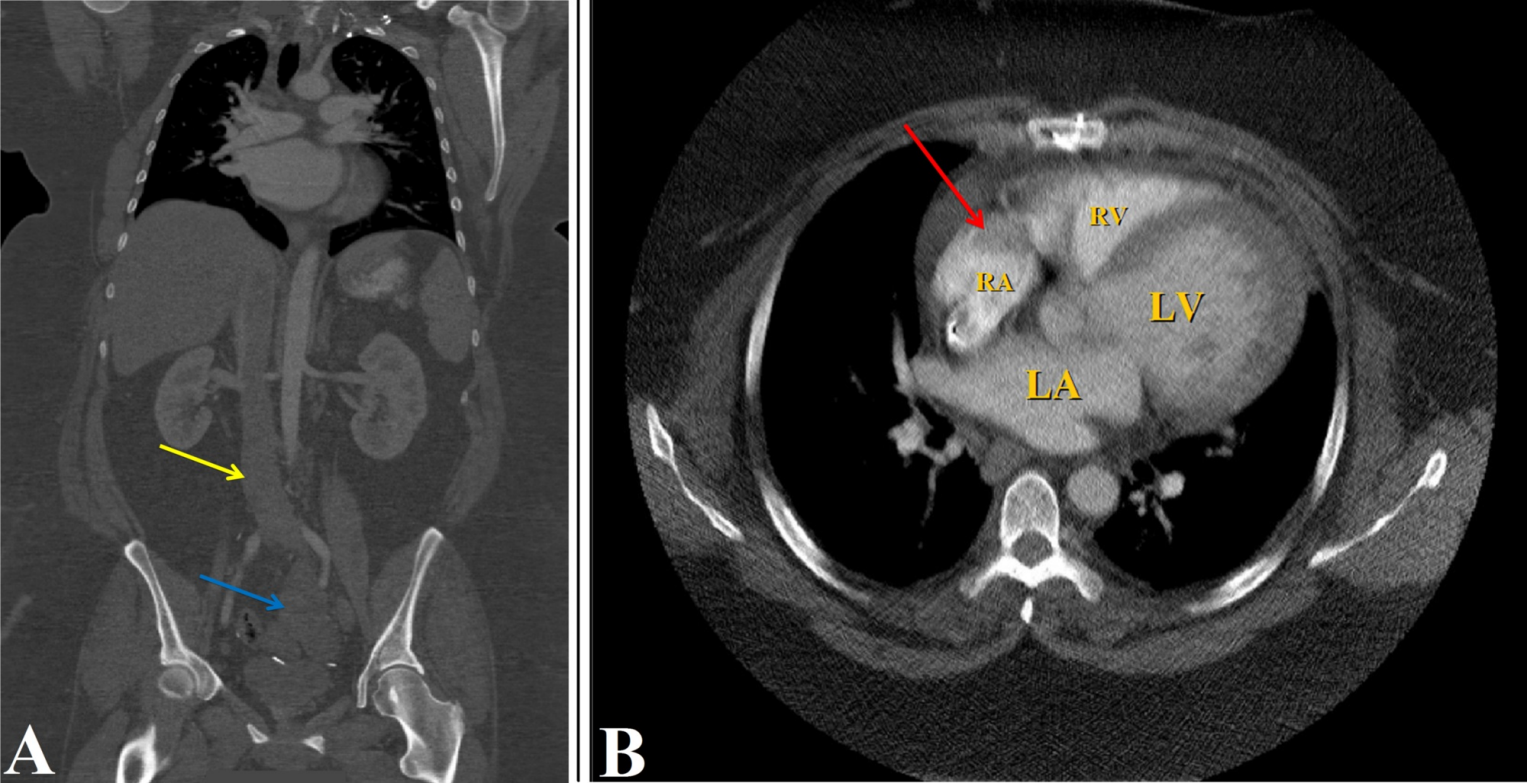
Contrast-enhanced CT abdomen and pelvis showing: (A) nonocclusive IVC thrombus extending to the intra-hepatic IVC into the iliac veins (yellow arrow), and left adnexal mass (blue arrow); (B) thrombus in the right atrium (red arrow). IVC, inferior vena cava; LA, left atrium; LV, left ventricle; RA, right atrium; RV, right ventricle

**Figure 2:**
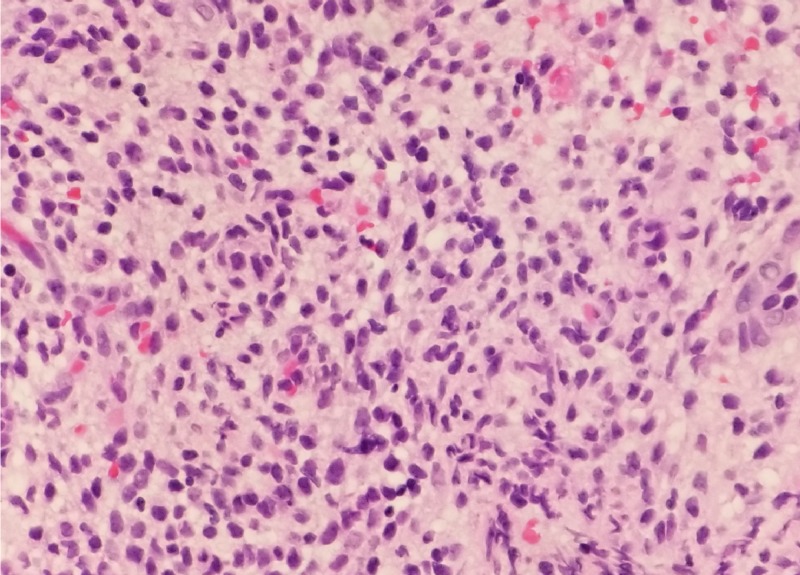
Tumor sample showing intravascular proliferation of leiomyoma consistent with IVL (H&E, x200). IVL, intravascular leiomyomatosis

Two years after the hysterectomy, the patient was sent to the ED from the cardiology clinic for dyspnea, dizziness, and multiple episodes of syncope. Physical examination was within normal limits except for the presence of jugular venous distension, and irregular heart rate and rhythm where electrocardiogram (EKG) showed atrial fibrillation and transthoracic echo (TTE) reported nonischemic cardiomyopathy with a left ventricular ejection fraction (LVEF) of 20%-25% and a globular mass measuring 4.0 cm x 3.5 cm, almost filling the entire right atrium. Further workup, including abdominal ultrasound, revealed an enlarged inferior vena cava (IVC) with an intraluminal thrombus and occlusive portal vein thrombus causing absent flow consistent with Budd-Chiari syndrome. CT scan of the abdominal pelvis reported an extensive 5.7 cm x 4.7 cm IVC thrombus extending contiguously from the right mid external iliac vein and the left common iliac vein through the IVC and into the right atrium, in addition to a lobulated 12.0 cm pelvic mass (Figure 1A, B). MRI of the abdomen and pelvis with and without contrast revealed a prominent solid, avidly enhancing portion within the sizeable pelvic mass. The patient underwent right atrial, IVC, and bilateral iliac tumor thrombus resection by a team of cardiothoracic and vascular surgeons. A follow-up CT angiogram of the chest with contrast reported no residual thrombus. Subsequent resection of pelvic mass reported spindle-shaped cells with degeneration consistent with IVL (Figure 2).

 

**Figure 3:**
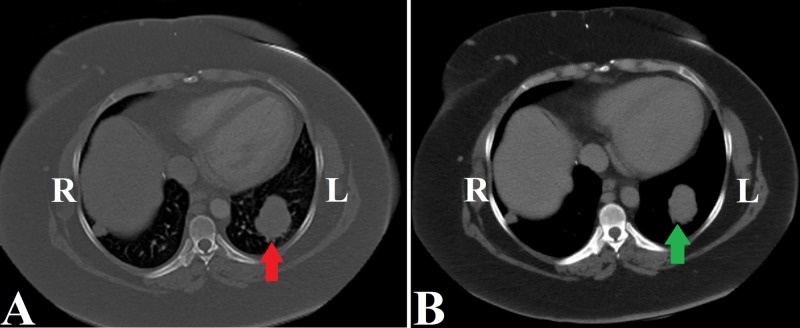
Chest CT showing: (A) multiple pulmonary nodules with the largest nodule measuring 4.7 cm x 3.8 cm (red arrow); (B) reduced size of the largest nodule to 4.1 cm x 3.1 cm upon follow-up (green arrow). R, right; L, left

A few months later, the patient also underwent an implantable cardioverter-defibrillator (ICD) placement for atrial fibrillation, and the patient's cardiac and functional status further improved, and she was able to get back to work. Two years later, the patient started complaining of an increasing lower extremity swelling, and dyspnea on exertion. A chest CT scan reported new pulmonary nodules (Figure 3A). 

Transjugular IVC mass biopsy performed, and pathology reported findings consistent with BML given the neoplasm exhibited staining with antibodies directed against S-100 and smooth muscle actin, without significant reactivity for antibodies directed against cytokeratins AE1/AE3 with ER+/PR+ on immunohistochemical stains. 

In light of her progressive disease, and worsening LVEF to 15%-20% upon preoperative evaluation, a multidisciplinary team recommended medical treatment consideration, and the patient was commenced on a new regimen of leuprolide and letrozole. While on this treatment, her abdominal mass and lung nodules were considered stable by RECIST 1.1 criteria (9.2% decrease in total tumor burden size), and she has had no new symptoms five years after initial diagnosis (Figure 3B). 

Molecular profiling

**Figure 4:**
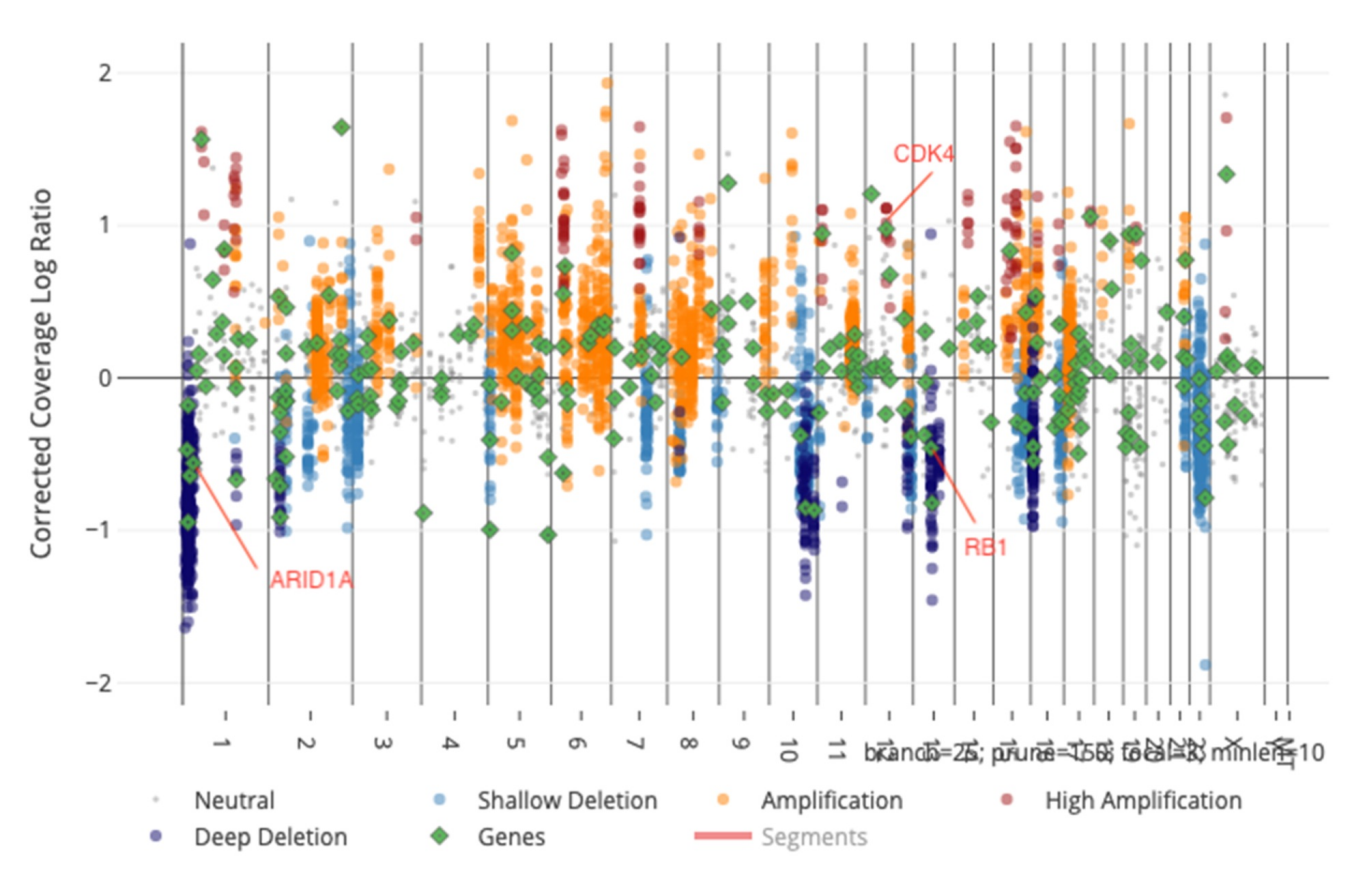
A spectrum of tumor cell-related genomic expression in our patient differentially showing a CDK4 oncogene copy number gain and copy number loss for ARID1A and RB1 tumor suppressor genes. CDK4, cyclin-dependent kinase 4; ARID1a, AT rich interactive domain 1A; RB1, retinoblastoma-1

Molecular profiling of the tumor showed amplification of cyclin-dependent kinase 4 (CDK4), JUN, and MCL1, and loss of AT-rich interaction domain 1A (AR1D1A), RB transcriptional corepressor 1 (RB1), SUFU, and hepatocyte nuclear factor 1-alpha (HNF1A) (Figure 4). 

## Discussion

Uterine leiomyoma (fibroid) is a common benign smooth muscle tumor in reproductive-age women. It has a spectrum of rare metastatic extrauterine variants, including IVL and BML. IVL characterized by benign smooth muscle tumor cells invade the uterine, pelvic veins, the vena cava, and may extend to the right heart. It is usually benign and susceptible to resection, but it can be fatal from resultant venous obstruction or cardiac involvement [6-8]. An additional uncommon extra-uterine smooth muscle infiltration variant is BML, which can be idiopathic or might occur following instrumentation for uterine leiomyoma, most commonly total hysterectomy [8]. Its controversial origin and the mystery of its pathogenesis made it even more challenging, but the vascular spread is the most widely accepted hypothesis [9-10]. It mostly affects the lungs in patients with a history of uterine leiomyomas [11-12].

On the contrary to aggressive metastatic leiomyosarcoma, BML has been characterized by low mitotic counts, slow-growth, and thus, their size, number, and clinical symptoms usually remain stable unless they are diagnosed late because of symptoms from tumor size. Treatment of pulmonary BML depends on the patient’s age, disease extent, and the estrogen receptor (ER) status; it may include hysterectomy with oophorectomy, debulking surgery and/or the use of GnRH analogs, aromatase inhibitors, selective ER modulators, or chemotherapy to decrease tumor burden in conditions that are unamenable for surgery [10, 13-15]. 

Our genomic analysis of the tumor sample identified multiple gene alterations (Table 1) of those, CDK4 gene, which regulates cycle G1 phase checkpoint. CDK activity has been previously identified as a potential biomarker of malignant phenotype [16]. Furthermore, the tumor suppressor gene ARID1A controls cell growing and division, the RB1 gene regulates cell survival, apoptosis, and differentiation, the SUFU gene regulates cellular proliferation, the proto-oncogene JUN signals cell division, the MCL1 gene inhibits apoptosis and promotes cell survival, and the homeobox gene HNF1A is expressed in organs of endodermal origin including liver, pancreas, and intestines [15].

**Table 1:** Genomic alterations identified in the tumor sample from our patient. CDK4, cyclin-dependent kinase 4; MCL1, myeloid cell leukemia-1; ARID1a, AT-rich interactive domain 1A; RB1, retinoblastoma-1; HNF1A, hepatocyte nuclear factor 1-α; SUFU, suppressor of the fused gene

Gene	Alteration	Function	Association with cancer progression
CDK4	Copy number gain	Oncogene	+
MCL1	Copy number gain	Oncogene	+
JUN	Copy number gain	Oncogene	+
ARID1A	Copy number loss	Tumor suppressor gene	+
RB1	Copy number loss	Tumor suppressor gene	+
HNF1A	Copy number loss	Tumor suppressor gene, transcription factor	+
SUFU	Copy number loss	Tumor suppressor gene, and a negative regulator of hedgehog signaling	+

Carcinogenesis is a dynamic multistep process involving the progressive accumulation of genetic and epigenetic alterations that ultimately transform normal cells to neoplastic cells. Molecular alterations that drive malignant transformation and progression, often involve oncogenes and tumor suppressor genes. In this study, the next-generation sequencing findings of amplification of multiple oncogenes and loss of tumor suppressor genes suggest molecular evidence of malignant behavior of uterine leiomyomas. Further larger studies may help further advance our understanding of BML and better targeted therapeutic approaches.

## Conclusions

This report describes a case of BML, for which molecular profiling of a tumor biopsy revealed several novel genomic amplifications that have not been previously implicated in BML. These findings propose a novel molecular association with malignant expression in patients with uterine leiomyomas. Future research is needed to help improve our knowledge about this tumor's behavior and explore new patient-specific therapeutic targets.
